# Self-Management of Hip Osteoarthritis Five Years After a Cycling and Education Treatment Pathway

**DOI:** 10.3390/healthcare8010037

**Published:** 2020-02-12

**Authors:** Thomas W. Wainwright, Louise C. Burgess, Tikki Immins, Robert G. Middleton

**Affiliations:** 1 Orthopaedic Research Institute, Bournemouth University, Bournemouth BH8 8EB, UK; lburgess@bournemouth.ac.uk (L.C.B.); timmins@bournemouth.ac.uk (T.I.); rmiddleton@bournemouth.ac.uk (R.G.M.); 2 The Royal Bournemouth and Christchurch Hospitals NHS Foundation Trust, Bournemouth BH7 7DW, UK

**Keywords:** hip, osteoarthritis, physical therapy, total hip replacement (THR), exercise, cycling

## Abstract

The Cycling against Hip Pain (CHAIN) programme is a six-week cycling and education treatment pathway for people with hip osteoarthritis. Preliminary results demonstrated significant improvements in clinical and patient-reported outcome measures following the course. Whilst the benefits of exercise for osteoarthritis are often reported in the short term, less is known about the long-term effects for this patient group. This study explores whether participants continued to self-manage their hip osteoarthritis five years after completing the course. A cross-sectional survey was conducted to collect data from participants who completed the CHAIN programme between October 2013 and February 2015 (*n* = 96). Questionnaires were sent by post in April 2019, and then non-responders were followed up again four weeks later. Eighty-three (87%) participants responded to the survey. Five years (range 4–6) after completion of a six-week cycling and education programme, 37 (45%) participants had not returned to their general practitioner for further treatment of their hip pain, and 47 (57%) had not pursued surgical intervention. All participants were still engaged in at least one physical activity per week and many reported that they had purchased a bike (29%), joined a gym (30%) or cycled regularly (indoor cycling 25%, outdoor cycling 24%). Eighty (96%) participants reported an increase in knowledge of self-managing their symptoms. The findings from this study suggest that many patients are motivated to self-manage their hip osteoarthritis, five years following a six-week cycling and education treatment pathway that encourages lifestyle change.

## 1. Introduction

Osteoarthritis ranks highly among contributors to global disability and pain [1], and prevalence is projected to increase as the population ages [2]. In the United Kingdom (UK), an estimated 2.46 million (10.9%) adults aged over 45 have osteoarthritis of the hip [3]. Pain and loss of function are the predominant symptoms that lead to treatment, including non-pharmacological, pharmacological, and surgical approaches [4]. The eventual treatment for end-stage hip osteoarthritis is joint replacement surgery, which is a very successful operation in terms of clinical outcomes [5,6,7] and cost-effectiveness [8,9]. However, surgery still presents major risks and complications such as dislocation, blood clots and infection [10]. In addition, whilst the survivorship of hip prosthesis continues to improve, wear is unavoidable, and so for patients undergoing surgery at a young age, a revision procedure will also likely be required. As disease-modifying options for end-stage osteoarthritis are limited and expensive, encouraging patients to self-manage their symptoms in the early stage of the disease process may have a substantial long-term benefit [11].

### The Cycling against Hip Pain (CHAIN) Programme

For patients with mild to moderate hip osteoarthritis in the UK, the National Institute for Health and Care Excellence (NICE) guidelines, in line with other international guidelines [12], recommend a combination of education and advice, exercise (aerobic and local muscle strengthening) and weight loss where appropriate as treatment [13]. Hence, in 2013, The Cycling against Hip Pain (CHAIN) programme was conceived as a conservative treatment pathway for patients presenting with hip osteoarthritis [14]. The aims of the programme were to encourage the self-management of symptoms, to reduce pain, and to improve functional ability through a six-week programme of education and static cycling sessions. It was anticipated that if patients were able to successfully manage their condition, the need for hip replacement could be delayed, or even avoided. Participants were recruited for the programme through referral from their general practitioner (GP). Participants attended a local leisure centre once a week for a thirty-minute education session, delivered by a physiotherapist, followed by thirty minutes of indoor cycling led by an exercise instructor. The sessions were progressive and supplemented with home exercises. Education topics included the benefits of exercise for osteoarthritis, diet and nutrition, pain relief, pacing of activities and lifestyle change.

The short-term results, collected via an assessment completed by participants after undertaking the programme (*n* = 96), have previously been published and found statistically and clinically significant improvements in patient-reported outcome measures (Oxford Hip score, Hip Disability and Osteoarthritis Outcome Score (HOOS) function, EQ5D-5L utility, EQ5D visual analogue scale (VAS) and pain on weight bearing) and objectively measured function (sit-to-stand and timed up and go scores) immediately after the intervention [14]. Many participants, including those with complex comorbidities [15], also reported psychological benefits, including increased confidence in managing their own hip pain and an increase in motivation to exercise. Whilst the benefits of exercise for osteoarthritis are often reported in the short term (either straight after the intervention or at up to one year follow up) [16,17,18,19,20], less is known about the longer-term sustainability of exercise for this population. To follow the initial published results, this survey examines how participants have managed their condition five years after undertaking the CHAIN programme.

## 2. Materials and Methods

A cross-sectional survey was posted in April 2019 to the 96 patients who had completed the pilot CHAIN programme between October 2013 and February 2015 [14]. Participants who were referred onto the course but did not complete it were not invited to take part in the survey.

### 2.1. Ethical Considerations

The National Health Service (NHS) Health Research Authority decision tool [21] and Research Department at the local NHS hospital confirmed that ethical approval was not required as this study was an evaluation of a previously delivered service. In keeping with good practice, the principles outlined in the Declaration of Helsinki [22] were followed. In addition, this article was guided by the STrengthening the Reporting of OBservational Studies in Epidemiology (STROBE) statement [23], which can also be used to report cross-sectional surveys [24], and recommendations for the conduct and reporting of survey research [25].

### 2.2. Data Collection

A cover letter clearly explained to the participant why they were being contacted, provided details of the lead researcher and asked the participant if they would complete a questionnaire (Supplementary materials Figure S1). The questionnaire was sent to all 96 participants who completed the CHAIN programme between 2013 and 2015. The tool incorporated dichotomous, multiple choice, and one open-ended question in order to collect details on the how the participant had managed their condition following the CHAIN programme, including any further treatment they had received for their hip pain. After a period of four weeks had passed, a follow up letter and questionnaire was sent to those who had not responded, in order to encourage a reply. Participants were supplied with a stamped addressed envelope and an email address so that they could easily return the questionnaire by either post or email. Questionnaires were labelled with an individual identification code but did not contain any personally identifiable data. Once the questionnaires were returned, the answers were inputted into a secure Excel spreadsheet (Microsoft UK, Reading, UK). The data collection period closed twelve weeks after the initial questionnaire had been sent to allow for a timely analysis.

### 2.3. Data Analysis

Descriptive statistics were used to summarise the characteristics and responses of the included sample. Data from the open-ended question were thematically analysed using an inductive approach to identify key themes [26]. First and second order themes were independently identified by two researchers (L.C.B and T.I) and any discrepancies between findings were resolved and refined through discussion with the research team.

## 3. Results

Of the 96 participants included in the sample, 83 (87%) took part in the survey. The characteristics of these participants at the time of referral to CHAIN are shown in Table 1 and are representative of the original cohort [14]. Thirteen participants did not respond to the letter that was sent and not all participants who returned their questionnaire completed every question. The mean age of the responders at time of analysis was 67.35 ± 8.59 years.

### Treatment for Hip Pain

Five years following completion of the course, thirty-seven (45%) participants had not returned to their GP for further treatment of their hip pain (Table 2). Thirty-six (43%) participants had undergone total hip replacement surgery for their originally affected hip and one participant (1%) had a hip replacement on their originally non-affected side. Forty-seven participants (57%) had not pursued surgical intervention for their originally affected hip. Seven participants (8%) had pursued further physiotherapy treatment and three (4%) had been admitted to hospital for an intraarticular injection under x-ray control into their hip joint. For those who did go on to require surgical intervention at the local NHS hospital (*n* = 27), the average time from completion of CHAIN until surgery was 26 months (range: 1–54).

Participants report an increase in knowledge (96%) and ability (93%) to self-manage hip pain following completion of the CHAIN programme. Twenty-four participants (29%) purchased a bike after completing CHAIN and 25 (30%) joined a gym. Only four (5%) participants were still in contact with someone they were on the CHAIN programme with. Ninety percent of responders stated that they would consider recommending the programme to a friend (82% yes, 8% maybe), and 59% were interested in participating again (40% yes, 19% maybe) (Table 3).

**Figure 1:**
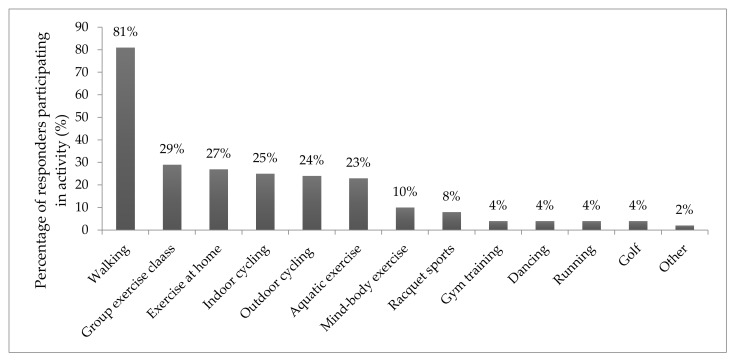
Activity participation at five year follow up.

At the time of data collection, all participants were engaging in at least one physical activity once a week (Figure 1). Walking was the most frequent activity reported, with 67 (81%) responders reporting that they walk at least once a week. Group exercise classes (29%) and exercise at home (27%) were also popular amongst responders, followed by both indoor and outdoor cycling (25% and 24% respectively). Aquatic exercise (swimming and aqua aerobics), mind-body exercise (yoga, Thai chi and Pilates) and racquet sports (badminton, tennis and table tennis) followed less frequently. 

Participants mostly answered the final question (question 12: please use this space to write down any other comments you have about the CHAIN programme) by describing benefits of the programme, offering suggestions for improvements, or giving details on constraints to participation in physical activity (Table 4). Not all participants answered this question, and where more than one theme existed in participant response, their answer was separated into the appropriate number of themes.

Benefits of the programme could be categorised into: (1) increased participation in physical activity levels, (2) physical benefits, (3) knowledge benefits and (4) social benefits. The increased physical activity theme was applied when the responder had described exercise behaviour change or engagement in a new activity following completion of CHAIN, for example: “Since CHAIN, I have bought a spinning bike and aim to spin daily for about 30 minutes. I practice this amount to about five times a week. When I saw [the Professor] in July 2014, I couldn’t walk without pain. Now I can walk for a whole day [and] we cycle on holidays. CHAIN has been a huge help to me.”

Physical benefits reported included weight loss, pain relief, muscle strengthening, and improved mobility and fitness. For example, “the programme was of good benefit as it improved my mobility, enabled me to lose weight and improved my leg muscles which all contributed to lessened pain.” The increased knowledge theme was applied when responders mentioned that they had learnt something new or had gained the confidence to exercise again. Social benefits were reported in 6% of these responses and included comments on enjoying the environment and meeting others with similar health conditions.

The “constraints” theme describes barriers or perceived constraints that complicated participation in CHAIN or have prevented participation in physical activity after CHAIN. These included other health problems and environmental barriers, such as a lack of accessible facilities. Seven participants (8%) offered suggestions as to how to improve the CHAIN programme. These were largely organisational or logistical, such as running CHAIN on a regular basis at a local gym, or turning the music down in the spin class. Suggestions for additional topics for the education component of the programme were also offered, for example, more information on yoga and Pilates.

Eight participants (10%) used this question to highlight that the programme had not sufficiently reduced their hip pain to prevent further intervention. Some responders mentioned that whilst they had felt they needed surgery, they had benefitted from cycling before and after their operation: “this programme was so beneficial to me just before my hip replacement operation. It improved my muscle mass and helped me to gain strength before I went in for surgery. I continued to cycle afterwards as I found it the best non-impact exercise for my recovery.”

## 4. Discussion

Recent guidelines recommend structured, land-based exercise programmes and education on arthritis as core treatment for the non-surgical management of hip osteoarthritis [27]. Whilst the benefits of exercise for osteoarthritis are often reported in the short term (either straight after the intervention or at up to one year follow up) [16,17,18,19,20], less is known about the longer-term sustainability of exercise for this patient group. This survey is the first to evaluate the CHAIN programme five years after completion and provides encouraging results for the long-term effectiveness of a six-week cycling and education intervention to help manage symptoms conservatively. However, as we did not repeat the validated questionnaires (such as the Oxford Hip Score, HOOS and VAS pain) that were outcome measures of the CHAIN programme, we cannot objectively compare symptoms five years from completion of the course.

Nonetheless, this survey suggests that at five year follow up, over half of the participants who completed CHAIN are still choosing to use exercise and self-management techniques to manage their hip pain. Importantly, taking part in the CHAIN programme appeared to encourage new or sustained participation in physical activity, including walking (81%), group exercise classes (29%), exercise at home (27%), indoor cycling (25%), outdoor cycling (24%) and aquatic exercise (23%). However, as this survey did not collect data on duration and frequency of exercise, there is no further information available on exercise dosage. Whilst some responders report perceived barriers or constraints to taking part in physical activity (such as a lack of suitable facilities or other health problems), all participants listed at least one activity for this question. Furthermore, 24 participants (29%) purchased either an indoor or outdoor bike (47% already owned a bike) and 25 participants (30%) joined a gym (12% were already a member). In addition to the important musculoskeletal benefits of exercise, these results are encouraging given the higher cardiovascular risk in patients with osteoarthritis due to inactivity [28], and the multiple benefits of exercise on all long-term chronic conditions usually faced by this age range of patients [29].

Exercise-therapy has been reported as cost effective and clinically effective for patients with osteoarthritis of the hip or knee, due to cost savings gained through reduced healthcare and raised productivity [30]. Five years after referral to CHAIN, 37 participants (45%) had not returned to their GP for further treatment of their hip pain and 47 (57%) had not pursued surgical intervention on their originally affected side. In addition, only one participant had received surgical intervention on their contralateral hip. For those who did go on to require surgical intervention at the local NHS hospital (*n* = 27), the average time from completion of CHAIN until surgery was 26 months (range: 1–54). However, there were a small number of patients who were referred to CHAIN despite already being listed for hip replacement surgery. Hence, they had a personal goal of preparing themselves for surgery, rather than avoiding it. In addition, as we did not measure the progression of osteoarthritis for these patients, and the pathogenesis of the disease is a complex and non-linear process [23], we cannot be certain that these patients would have required surgical intervention without participating in the CHAIN programme. It should also be noted that as only 75 out of 96 of the participants had a clinical diagnosis of osteoarthritis at point of referral onto CHAIN, it cannot be assumed that all participants would become candidates for joint replacement in the future.

Whilst the short-term physical improvements are important, the eventual aim of the CHAIN programme is to encourage participants to self-manage their symptoms by making specific, overt changes to their lifestyle, which are maintained long term without relapse. Our qualitative findings suggest that for many patients, learning that it is safe and beneficial for a person with osteoarthritis to exercise, and meeting others with a similar condition, are substantial contributors to an increase in physical activity participation. Our findings are consistent with a recent Cochrane review on exercise interventions and patient beliefs for people with osteoarthritis [31]. The review found that patient beliefs about chronic pain shape their attitudes and behaviours about how to manage their pain. Hence, those who are unsure on what exercise they should and should not do, avoid activity due to fear of causing harm. Providing reassurance and clear advice about the value of exercise in controlling symptoms, and opportunities to participate that are considered enjoyable and relevant, are thought to lead to greater exercise participation [31]. In addition, it is thought that group-mediated delivery can foster change through group cohesion, modelling, mastery experience and increased self-efficacy, supporting the use of group exercise, over individual interventions for this patient group [32].

### Limitations

The lack of control group and small sample size are clear limitations of this study. Despite being suited to descriptive analysis, cross sectional studies are limited to giving results for a given period of time, with no consideration of the sequence of events occurring before or after. As the participants who did not complete the course were excluded from this analysis, an element of selection bias is likely to exist, whereby those who completed the course were already aware of the benefits of physical activity and motivated to exercise. Whilst the questionnaire and cover letter were designed to elicit a neutral response, it is likely that an element of response bias exists in the results, whereby responders felt they needed to report positive experiences to the researchers. As previously mentioned, the absence of objective, validated questionnaire data precludes the comparison of symptoms five years following the course. Finally, as only 83 out of 96 participants returned their questionnaire, these results only represent a sample of the participants who completed the CHAIN programme between 2013 and 2015. However, a response rate of 60% in survey research has previously been reported as the threshold of acceptability [33].

## 5. Conclusions

Whilst the research evidence encouraging exercise and education programmes for the conservative management of osteoarthritis is increasing, less is known about the long-term impact of these interventions on patient behaviours. The findings from this study suggest that many patients are motivated to self-manage their hip osteoarthritis, five years following a six-week cycling and education treatment pathway that encourages lifestyle change. Future work in this area will involve delivering a randomised controlled trial to compare the outcomes of the CHAIN programme to standard care physiotherapy, in order to obtain further evidence on its impact (study ID: ISRCTN19778222). Results from this trial will contribute to more comprehensive recommendations for the management of hip osteoarthritis.

## Figures and Tables

**Table 1:** Characteristics of survey responders at time of referral to Cycling against Hip Pain (CHAIN).

Characteristic		Frequency (%)
Gender *n* (%)	Male	38 (46%)
	Female	45 (54%)
Primary diagnosis *n* (%)	No diagnosis	10 (13%)
	Osteoarthritis	64 (80%)
	Rheumatoid arthritis	1 (1%)
	Post Traumatic	1 (1%)
	Other	4 (5%)
	Not stated	3 (4%)
Body Mass Index (BMI) *n* (%)	<18.5 (underweight) 18.5–24.9 (normal)	1 (1%) 23(28%)
	25.0–29.9 (overweight)	26 (31%)
	30.0 and over (obese)	20 (24%)
	Not stated	13 (16%)
Age mean (SD)		62.66 (8.74)
Baseline Oxford Hip score mean (SD)		33.51 (7.81)

**Table 2:** Treatment for hip pain following completion of the CHAIN programme.

Question	Answer	Frequency (%)
General practitioner (GP) visit for hip pain	Yes No	46 (55%) 37 (45%)
Treatment to affected hip	No treatment Total hip replacement Unilateral Bilateral Physiotherapy Intra-articular injection	37 (45%) 36 (43%) 34 (94%) 2 (6%) 7 (8%) 3 (4%)
Hospital	Local NHS hospital Local private hospital Elsewhere	27 (75%) 7 (19%) 2 (6%)
Year of hip replacement	2014 2015 2016 2017 2018 2019	5 (14%) 8 (22%) 6 (17%) 8 (22%) 7 (19%) 2 (6%)
Treatment to non-affected hip	Total hip replacement Intra-articular injection Pain killers No treatment	1 (1%) 1 (1%) 2 (2%) 79 (95%)

**Table 3:** Participant experience of CHAIN.

Question	Answer	Frequency (%)
Did undertaking the CHAIN programme increase your knowledge of self-managing your hip pain?	Yes A little No Did not answer	68 (82%) 12 (14%) 2 (2%) 1 (1%)
After the programme, did you feel that you were able to self-manage your hip pain?	Yes A little No Did not answer	54 (65%) 23 (28%) 5 (6%) 1 (1%)
Since completing CHAIN, have you purchased a bike?	Yes Indoor Outdoor No Already owned a bike	24 (29%) 17 (71%) 7 (29%) 20 (24%) 39 (47%)
Since completing CHAIN, have you joined a gym or leisure centre?	Yes Already a member No Did not answer	25 (30%) 10 (12%) 46 (55%) 2 (2%)
Are you still in contact with anyone you were on the course with?	Yes No	4 (5%) 79 (95%)
Would you recommend the course to a friend?	Yes Maybe No Did not answer	68 (82%) 7 (8%) 2 (2%) 6 (7%)
Would you be interested in completing the CHAIN programme again?	Yes Maybe No Did not answer	33 (40%) 16 (19%) 27 (33%) 7 (8%)

**Table 4:** Themes in comments from survey responders.

Theme	Frequency (%)
Increased participation in physical activity	20 (25)
Physical benefits	14 (18)
Knowledge benefits	14 (18)
Constraints	11 (14)
Limited effects	8 (10)
Suggestions for improvement	7 (9)
Social benefits	5 (6)
Total	76

## Supplementary Materials

The following are available online at https://www.mdpi.com/2227-9032/8/1/37/s1, Figure S1: Questionnaire.
