# Supplementary material for: Self-Management of Hip Osteoarthritis Five Years After a Cycling and Education Treatment Pathway
Source: Healthcare (Basel). 2020 Feb 12;8(1):37. doi: 10.3390/healthcare8010037 (PMC7151257; doi:10.3390/healthcare8010037)
Supplement: Supplementary file 1 [file healthcare-08-00037-s001.pdf]

Supplementary Material 1: Questionnaire

The CHAIN (Cycling Against Hip Pain) Programme

We would like to know how you are doing after completing the CHAIN programme which ran in Bournemouth between 2013 and 2015 for the treatment of hip pain, and would be grateful if you could complete the following questionnaire **in regard to the hip you were being treated for at the time of referral (i.e. affected hip).** If there are any questions that seem unclear, please contact [lead researcher] on [phone number/email address].

1. Did undertaking the CHAIN programme increase your knowledge on self-managing your hip pain? *(Please circle)*

a. Yes   b. A little   c. No

2. After the programme, did you feel that you were better able to self-manage your hip pain?

a. Yes   b. A little   c. No

3. Since completing CHAIN, do you take part in any of the following activities at least once a week?
Please circle as many as applicable.

a. Outdoor cycling   b. Indoor cycling   c. Group exercise class   d. Walking

e. Running   f. Swimming   e. Exercise at home   g. Other (please state)

____

4. Since completing the CHAIN programme, have you purchased a bike?

a. Yes   b. No   c. I already owned a bike

If yes, what sort of bike did you purchase?

a. Indoor bike (static)   b. Outdoor bike

5. Since completing CHAIN, have you joined a gym or leisure centre?

a. Yes   b. No

6. Are you still in contact with anyone who you were on the CHAIN programme with?

a. Yes   b. No

7. Since being referred to the CHAIN programme, have you visited your general practitioner (GP) for further treatment of hip pain for your affected hip?
If both hips were affected, please specify which side was treated.

a. Yes b. No

If yes, how many times?____

8. Since being referred to the CHAIN programme, have you received any of the following treatments for your affected hip(s)?
If both hips were affected, please specify which side was treated.

a. Physiotherapy (please state how many sessions below) b. Steroid injection c. Hip replacement surgery d. Other (please state below)

If yes, which hospital were you treated at and what year were you treated?

Hospital:____________________________________**Year:**__________________________

9. Following the CHAIN programme, has your non-affected hip received any treatment for hip pain?
Do not complete if both hips were originally affected.

a. Yes b. No

If yes, please circle which treatment(s) you received:

a. Physiotherapy (please state how many sessions below) b. Steroid injection c. Hip replacement surgery d. Other (please state below)

---

10. Would you recommend the CHAIN programme to a friend?

a. Yes b. Maybe c. No

11. Would you be interested in doing the CHAIN programme again?

a. Yes b. Maybe c. No

12. Please use this space to write down any other comments you have about the CHAIN programme

Thank you for taking the time to complete this questionnaire. Once completed, please either scan and email it to [email address] or send it by post to: [postal address].
